# Exosome-Associated Gene Signature for Predicting the Prognosis of Ovarian Cancer Patients

**DOI:** 10.1155/2023/8727884

**Published:** 2023-01-23

**Authors:** Zihan Zhu, Rui Geng, Yixin Zhang, Jinhui Liu, Jianling Bai

**Affiliations:** ^1^Department of Biostatistics, School of Public Heath, Nanjing Medical University, Jiangning District, 101 Longmian Avenue, Nanjing 211166, China; ^2^Department of Gynecology, The First Affiliated Hospital of Nanjing Medical University, Nanjing, 210029 Jiangsu, China

## Abstract

**Background:**

The exosome is of vital importance throughout the entire progression of cancer. Because of the lack of effective biomarkers in ovarian cancer (OV), we intend to investigate the connection between exosomes and tumor immune microenvironment to verify that exosome-related genes (ERGs) can precisely forecast the prognosis of OV patients.

**Methods:**

First, 117 ERGs in The Cancer Genome Atlas (TCGA) dataset were recognized. Afterwards, the risk signature consisting of four ERGs with prognostic significance was built by univariate Cox, least absolute shrinkage and selection operator (LASSO), and multivariate Cox regression analysis. We also validated the risk signature by Kaplan-Meier analysis, receiver operating characteristic curve analysis and principal component analysis. Furthermore, gene set enrichment analysis was performed to compare the enrichment patterns between the two risk subgroups. The connections between the exosome-related gene risk score (ERGRS) and clinical features, immune infiltration, immune checkpoint-related genes, copy number variation, and drug sensitivity were explored. We also assessed the function of the ERGRS to forecast immunotherapeutic efficacy by immunophenoscore (IPS).

**Results:**

The high-risk group had a worse prognosis than the group with low risk. We verified that the established model possessed a relatively good prognostic value. Pathway enrichment analysis indicated that the genome-wide group with low risk was enriched in immune-related pathways. We discovered that resting dendritic cells and stromal scores were upregulated in patients with high risk in the TCGA and Gene Expression Omnibus (GEO) cohorts. Moreover, the expression of six common immune checkpoint inhibitor targets was assessed, which revealed that the expression levels of CD274 (PD-L1), PDCD1 (PD-1), and IDO1 in patients with high risk were lower than those in patients with low risk. Afterwards, the low-risk group had higher IPS across the four immunotherapies, implying that it had better effects of immunotherapies.

**Conclusion:**

Our study demonstrates that the exosome-related gene risk model is closely associated with immune infiltration. It can well forecast the prognosis of OV patients and guide the selection of immunotherapeutic strategies.

## 1. Introduction

Ovarian cancer (OV) is a common gynecological tumor around the world, consisting of about 4% of all new cancer cases in women. OV often occurs in older people, and over half of new cases are diagnosed in women after the age of 65 [1]. Due to lacking obvious early symptoms and effective diagnostic strategies, OV has the highest mortality among gynecological cancers [2]. Traditionally, conventional treatment for OV includes debulking surgery and platinum-based chemotherapy [2–4]. Nevertheless, due to relapse and chemoresistance, the current five-year survival rate for OV is approximately 47% [5]. Therefore, there is a need to further investigate the underlying mechanisms of OV progression and seek more effective biomarkers.

The exosome is an intraluminal vesicle with a diameter of 30 to 150 nm, generated by the budding of the endosomal membrane [6–8]. When first discovered in 1983 [8], it was recognized as a cellular waste to be disposed of [9]. In the study of biological functions, exosomes originating from tumors have been proved to take part in the swap of genetic data between basal cells and tumor cells, leading to the growth of abundant new blood vessels, which promote the occurrence, progression, invasion, and metastasis of tumors [6, 10–12]. On the other hand, some tumor-secreted exosomes also carry various immunosuppressive molecules [13], which can inhibit the proliferation of CD4^+^ and CD8^+^ T lymphocytes, or stimulate the differentiation of immunosuppressive cells, including regulatory T lymphocytes or myeloid cells [14–16]. Therefore, the exosome can mediate the immunosuppression of tumor-host cells and is closely connected with tumor immunotherapy.

In recent years, based on the immunomodulation formed by the interaction between the tumor immune microenvironment (TIME) and cancer cells [17, 18], several immunotherapies have been shown to obtain promising outcomes in treating tumors. As is demonstrated by numerous studies, tumor immune-infiltrating cells (TIICs) in TIME are critical for the therapeutic effect of immunotherapy and cancer progression [19]. In the past 20 years, immunotherapy has a rapid development, which revolutionizes the remedy for various cancers. Since OV is now generally considered an immunogenic tumor, advances in immunotherapy have offered new opportunities for treating OV [20–23]. Hence, finding new biomarkers to forecast the response to different immunotherapies is needed.

In the study, we mainly established and validated the exosome-related gene risk model (ERGRM) and utilized the nomogram to better predict the prognosis of patients. After that, by functional enrichment analysis, immune infiltration level analysis, and copy number variation analysis, the relationship between the exosome-related gene risk score (ERGRS) and TIME was deeply explored. Finally, the vital role of this model in guiding the selection of therapeutic strategies was illustrated by calculating the gene expression of important immune checkpoint inhibitors (ICIs) and drug sensitivity.

## 2. Materials and Methods

### 2.1. Data Acquisition

RNA-seq and clinical data of OV patients were downloaded from The Cancer Genome Atlas (TCGA) database (https://cancergenome.nih.gov/) and GSE9891 in the Gene Expression Omnibus (GEO) database (https://www.ncbi.nlm.nih.gov/geo/) as TCGA dataset and GEO dataset. Furthermore, normalization and removal of batch effects between the two datasets were performed by the “sva” *R* package [24]. In addition, we also downloaded 121 exosome-related genes (ERGs) from the ExoBCD database (https://exobcd.liumwei.org/). The TCGA dataset was split into the training group and the testing group with a 1 : 1 ratio, where the TCGA training group was employed to build the ERGRM to forecast the prognosis of OV patients. Afterwards, the prognostic power of the ERGRM was verified through the TCGA testing set, the entire TCGA dataset, and the GEO dataset.

### 2.2. Establishment of a Prognostic Risk Model

First, ERGs in the TCGA cohort were confirmed. In the training set, the univariate Cox regression analysis of overall survival was carried out to identify ERGs with potential predictive value. In addition, LASSO regression analysis was utilized to decrease redundant genes and prevent overfitting of the ERGRM [25]. Then, multivariate Cox analysis was then employed to determine the risk score, which was evaluated on the basis of the following method: risk score = ∑_*i*=1_^*n*^(Exp_*i*_∗Coe_*i*_), where Exp_*i*_ meant the ERG expression, and Coe_*i*_ represented the corresponding multivariate Cox regression coefficient.

### 2.3. Validation of a Risk Model

All samples were grouped into high-risk and low-risk subgroups by setting the median score of the dataset as the critical value. In order to contrast the differences in overall survival between patients in both risk groups, Kaplan-Meier survival analysis was made by the “survminer” *R* package. To assess the predictive power of the risk model, receiver operating characteristic (ROC) curves were given, and the area under the curve (AUC) was computed with the “survivalROC” *R* package. Principal component analysis was carried out by the “prcomp” function of the “stats” *R* package to evaluate the discriminative ability of the model for OV patients.

### 2.4. Correlation of Risk Score with Various Clinical Features

We explored the connections between the ERGRS and different clinic pathology features. Chi-square tests were used to test different ratios of survival status, age, tumor stage, tumor grade, therapy type, and breast cancer susceptibility gene 1 (BRCA1) type in both risk groups. Differences in risk scores for subgroups of the above clinical characteristics were also compared by Student's *t*-test. Moreover, different clinical characteristics were stratified, and Kaplan-Meier curves were then employed to assess the prognostic power of risk scores across different layers.

### 2.5. Establishing and Verifying a Predictive Nomogram

Age and tumor stage were also verified to be independent predictors based on univariate and multivariate Cox analyses. We evaluated the specificity and sensitivity of some predictors by the AUC, which verified the reliability of the combination of the ERGRS and clinical factors. Therefore, to extend the prognostic power of the exosome-related prognostic model, nomograms were constructed according to the risk score, age, and tumor stage [26]. In the nomogram, assigning a score to each parameter and calculating their total score reduce the ERGRM to a single numerical estimate of event probability. Finally, calibration curves were constructed to verify the predictive ability of the nomogram.

### 2.6. Gene Set Enrichment Analysis

In order to investigate the biological pathways related to the ERGRS, gene set enrichment analysis (GSEA) was conducted on the whole genome of different risk groups through GSEA software (https://www.gsea-msigdb.org/gsea/) [27]. C2, one of the nine major collections in the Molecular Signatures Database, was employed as the compared set. In addition, gene sets with a nominal *p* value <0.05 were considered significant.

### 2.7. Assessment of Immune Infiltration Levels

The CIBERSORT algorithm, single-sample gene set enrichment analysis (ssGSEA), and ESTIMATE were utilized to investigate the relationship between TIME and exosome risk scores. Immune cell infiltration was estimated and analyzed through the CIBERSORT algorithm (http://cibersort.stanford.edu/). The ratio of 22 immune-infiltrating cells in both risk groups was evaluated by CIBERSORT [28]. Using the fractions of various TIIC components in all samples assessed by CIBERSORT, Wilcoxon's tests were performed to compare differences in various TIICs in different groups. The relationships between the ERGRS and the infiltration level of immune cells were also analyzed by Pearson's correlation analysis. Furthermore, ssGSEA analysis was performed by the “GSVA” *R* package to assess the connections between risk score and immune cell function. Since immune cells and stromal cells are the two major nontumor constituents of the TIME, the stromal scores obtained by the ESTIMATE method were utilized to explore the connections between the proportion of stromal cells and the ERGRS [29].

### 2.8. Role of the Risk Score in Forecasting the Effect of Immunotherapy

First, the expression of various immune checkpoint genes in both risk groups was analyzed. Subsequently, six common targets of ICIs were identified, and the connections between the expression of target genes and the ERGRS were assessed through Pearson's correlation analysis. Immunophenoscore (IPS) was computed through *Z*-scores of four classes of genes connected with immunogenicity, which could quantify the immunotherapeutic response [30]. Therefore, we selected two targets of ICIs (CTLA4 and PD-1) that were closely related to the ERGRS and assessed the relationship between the ERGRS and immunotherapeutic response by IPS. The IPSs of patients were acquired from The Cancer Immunome Atlas.

### 2.9. Copy Number Variation Analysis

The Genomic Identification of Significant Targets in Cancer (GISTIC) algorithm was employed to find unusual regions [31]. We used custom settings on the base of GISTIC2.0. Thresholds of amplification and deletion, confidence level, and focal length cutoff were defined as 0.10, 0.90, and 0.50, respectively. In addition, regions with *q* value <0.25 were significantly abnormal regions with recurrent copy number variation [31]. We employed GRCh37 (hg19) as the human genome reference.

### 2.10. Drug Sensitivity Analysis

The NCI-60 database, containing 60 cancer cell line data, was explored through the CellMiner website (https://discover.nci.nih.gov/cellminer/). Gene expression status and *Z*-score for drug sensitivity were extracted online. Then, Pearson's correlation analysis was used to explore the connections between the four exosome-related prognostic gene expressions and the sensitivity of 216 FDA-approved drugs. Afterwards, we obtained relevant information from the Genomics of Drug Sensitivity in Cancer (GDSC) database (https://www.cancerrxgene.org/) and analyzed the significance of the difference in the half-maximal inhibitory concentration (IC50) between different risk groups by Wilcoxon's test.

### 2.11. Statistical Analysis

Statistical analyses in our study were performed by *R* software (version 4.0.5). Important predictors were assessed by univariate and multivariate Cox regression analysis. The prognostic efficiency of the ERGRM was evaluated through the ROC curve. Differences in overall survival between groups were calculated through Kaplan-Meier analysis. We examined differences between two groups of variables through Student's *t*-test and Wilcoxon's test. The connections between the two factors were explored through Pearson's correlation analysis. Related graphics were drawn by employing *R* packages such as “pheatmap,” “ggplot2,” “GGPUBR,” and “ggExtra.” *p* < 0.05 was defined as statistically significant.

## 3. Results

### 3.1. Establishment and Verification of an ERGRM

**Figure 1:**
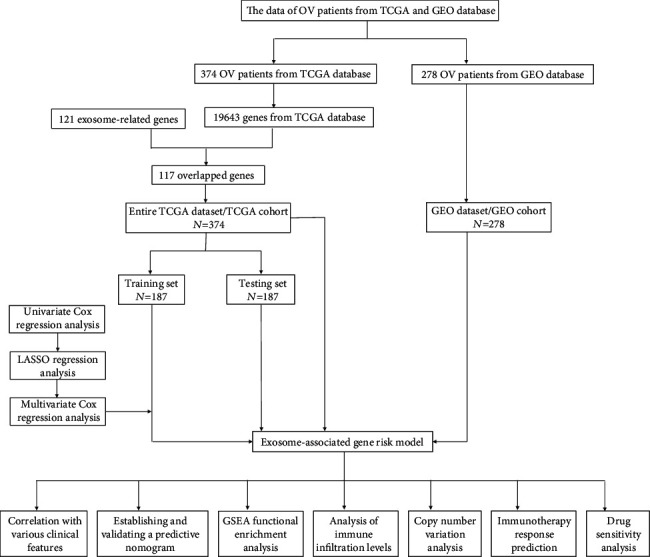
Flow chart of this study.

The figure below shows the research process of this study (Figure 1). An exosome-related gene set containing 121 genes involved in immune regulatory pathways was downloaded from the ExoBCD database, and 117 of them had expression values in the TCGA dataset (Supplementary Figure 1). TCGA dataset was split into the training set and testing set, and the ERGRM was established through TCGA training set. By univariate Cox regression analysis for preliminary screening, 5 of 117 ERGs were demonstrated to be related to the overall survival of patients, which included USF1, SNRPA1, ADAM10, PIGR, and MRPL15. To avoid model overfitting, LASSO analysis was carried out on these five genes, and four important prognostic genes were finally identified based on the minimum criteria (Supplementary Figures 2A, B). Then, an ERGRM was established according to the expression of the four genes and the regression coefficients derived from multivariate Cox regression analysis (Supplementary Figure 3), as follows: risk score = (−0.038 × expression level of USF1) + (−0.057 × expression level of SNRPA1) + (−0.019 × expression level of PIGR) + (−0.008 × expression level of MRPL15).

**Figure 2:**
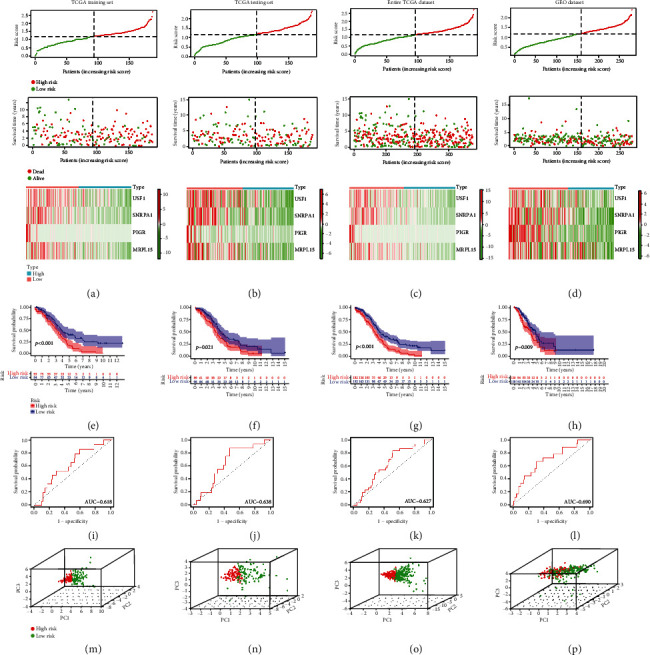
Prognostic analysis of exosome-related gene prognostic risk models in TCGA training set, TCGA testing set, entire TCGA dataset, and GEO dataset. (a–d) Distribution of risk scores, survival status, and expression of four prognostic genes in the four datasets. (e–h) In the four datasets, Kaplan-Meier analysis was used to compare the overall survival of patients in the high-risk and low-risk groups (*p* < 0.05). (i–l) ROC curves for the predictive power of prognostic risk models in the four datasets. (m–p) Plots of principal component analysis in the four datasets.

In the training set, low-risk patients always had longer survival times, lower risk of death, and higher expression levels of the four prognostic genes than high-risk patients (Figure 2(a)). This illustrated that different risk groups could discriminate the survival status and expression of four prognostic genes. Similarly, three other datasets revealed consistent results with TCGA training set, meaning that patients with high risk had worse prognosis than patients with low risk (Figures 2(b)–2(d)). At the same time, the results of the Kaplan-Meier analysis indicated that high-risk patients had lower survival rates than low-risk patients in all four cohorts (Figures 2(e)–2(h)). The ERGRM was validated to have good predictive accuracy through AUC values (AUC = 0.618, 0.638, 0.627, and 0.690 in the training set, testing set, entire TCGA dataset, and GEO dataset, respectively, Figures 2(i)–2(l)), and principal component analysis indicated that patients could be wholly categorized according to different risk groups (Figures 2(m)–2(p)). As was shown by the above results, the ERGRM was verified with good prognostic power.

### 3.2. Relationship between Risk Score and Various Clinical Features

In the TCGA dataset, the connections between the ERGRS and some clinic pathology characteristics (survival status, age, tumor stage, tumor grade, therapy type, and BRCA1 type) were analyzed. Supplementary Figure 4A displays that high-risk patients had higher percent weight of death status than low-risk patients, and that patients with death status tended to obtain higher risk scores (*p* = 0.0012). Supplementary Figure 4B indicates that elderly patients (age > 60) in the group with high risk had higher percent weight, and that elderly patients also had higher risk scores than nonelderly patients (*p* = 0.029). However, no significant connections were discovered between risk score and tumor stage, tumor grade, therapy type, or BRCA1 type (*p* > 0.05, Supplementary Figures 4C-F).

**Figure 3:**
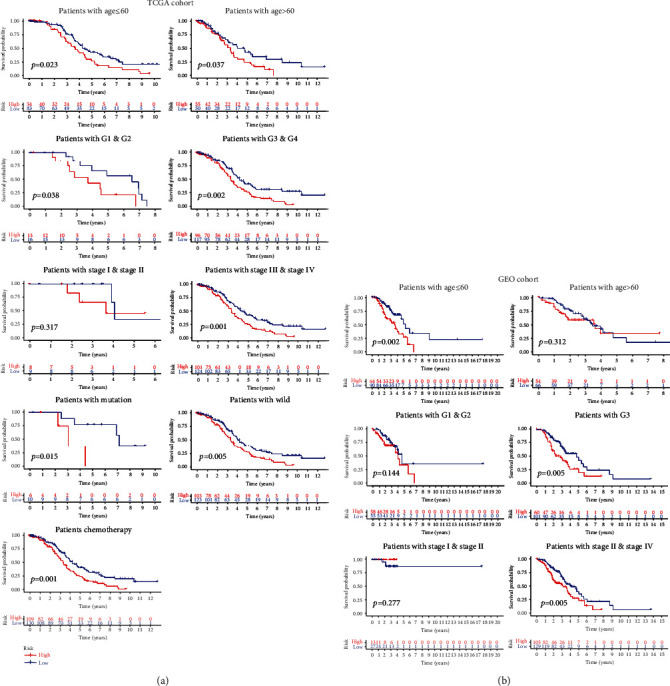
The ability of ERGRS to distinguish survival probability under different clinical characteristics. (a) In the TCGA cohort, Kaplan-Meier curves of different risk groups with different clinical characteristics, including age, tumor stage, tumor grade, BRCA1 type, and treatment type. (b) Kaplan-Meier curves of different risk groups with different characteristics of age, tumor stage, and tumor grade in the GEO cohort.

Afterwards, different clinical characteristics were stratified, and Kaplan-Meier analysis was employed to assess the prognostic ability of the ERGRS across different layers. According to Figures 3(a) and 3(b), risk scores achieved satisfactory prognostic identification in patients with age ≤ 60 years (*p* = 0.023), age > 60 years (*p* = 0.037), grades G1 and G2 (*p* = 0.038), grades G3 and G4 (*p* = 0.002), stages III and IV (*p* = 0.001), mutant-type BRCA1 (*p* = 0.015), wild-type RCA1 (*p* = 0.005), and chemotherapy (*p* = 0.001) in TCGA cohort. In the GEO cohort, risk scores achieved satisfactory prognostic discrimination in patients with age ≤ 60 years (*p* =0.002), G3 (*p* = 0.005) and stages III and IV (*p* = 0.005), and better survival rates appeared in patients with low-risk scores.

### 3.3. Establishment and Validation of a Predictive Nomogram

By univariate analysis, we found that the ERGRS was linked to overall survival in a significant way in the TCGA and GEO cohorts (*p* < 0.001, hazard ratio (HR) = 1.816 (95% confidence interval (CI), 1.369–2.410) and *p* = 0.005, HR = 1.851 (95% CI, 1.208–2.835)). Subsequently, by multivariate Cox regression analysis, the ERGRS was confirmed to be an independent predictor of OV patients in the TCGA and GEO cohorts (*p* < 0.001, HR = 1.750 (95% CI, 1.319–2.323) and *p* = 0.008, HR = 1.820 (95% CI, 1.168–2.834)). Likewise, age and tumor stage were also found to be independent prognostic predictors (Supplementary Table 1).

Next, we evaluated the predictive efficiency of the ERGRS for forecasting the prognosis of OV patients by AUC. AUC of the risk score was 0.624 in the TCGA cohort and 0.693 in the GEO cohort, and they were confirmed to be higher than those of some clinical features (Supplementary Figures 5A, B). However, when clinical factors and risk scores were combined, the AUC values of the combination were the highest in the TCGA and GEO cohorts, at 0.697 and 0.731, respectively (Supplementary Figures 5C, D). The above results again suggested that the ERGRS is an important prognostic predictor for OV patients, and the combination of other clinical features and the ERGRS is reliable.

**Figure 4:**
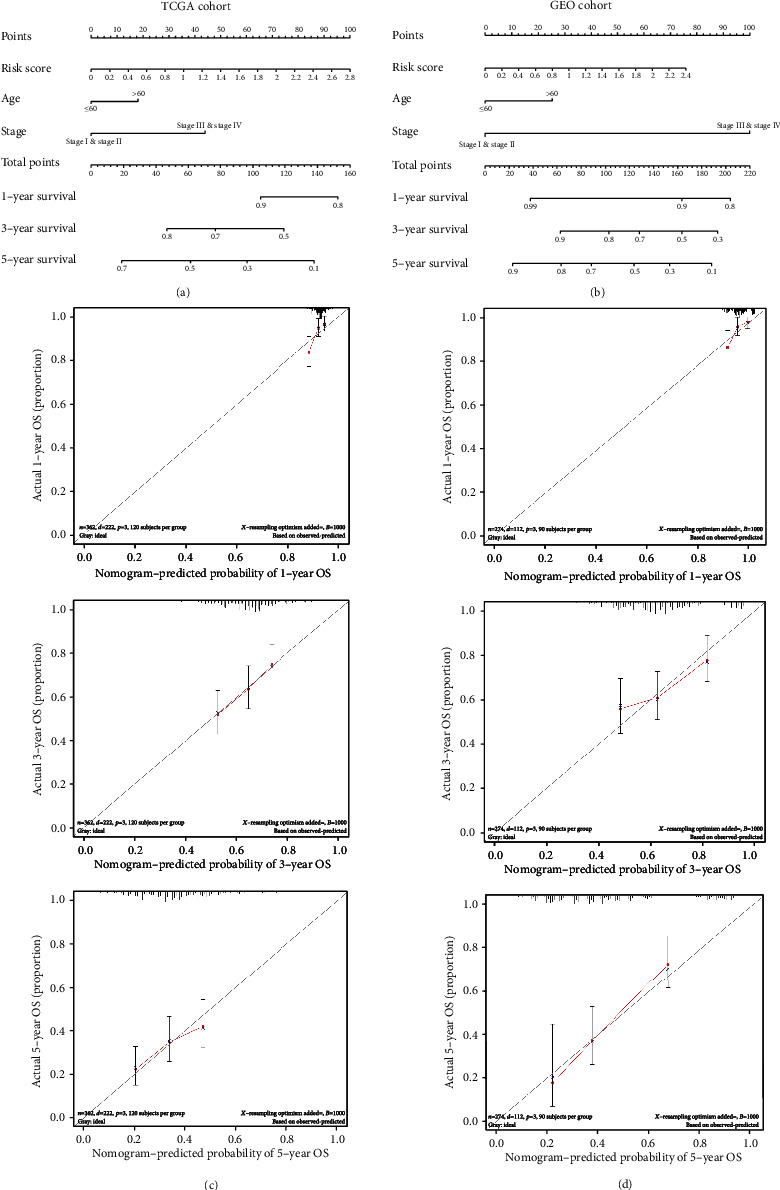
Construction and validation of the nomogram. (a, b) Nomograms of the TCGA and GEO cohorts were used to predict 1-, 3-, and 5-year survival probabilities in OV patients. (c, d) In the TCGA and GEO cohorts, calibration curves of nomograms showed the relationship between predicted and actual curves.

To expand the predictive performance of the ERGRM, nomograms were established on the basis of risk score, age, and tumor stage (Figures 4(a) and 4(b)). With a nomogram, we could forecast 1-, 3-, and 5-year survival probabilities. Calibration curves for 1, 3, and 5 years were established to verify the predictive performance of the nomogram (Figures 4(c) and 4(d)), revealing an ideal consistency between the prediction and reality.

### 3.4. Gene Set Enrichment Analysis

According to the results of GSEA, we discovered that the genome-wide group with high risk was shown to be enriched in the cancer-related KEGG pathway (Supplementary Figure 6A). Meanwhile, “primary immunodeficiency,” “intestinal immune network for IGA production,” “graft versus host disease,” “autoimmune thyroid disease,” and “allograft rejection” were mainly enriched in patients with low-risk scores (Supplementary Figure 6B).

### 3.5. Analysis of Immune Infiltration Levels

**Figure 5:**
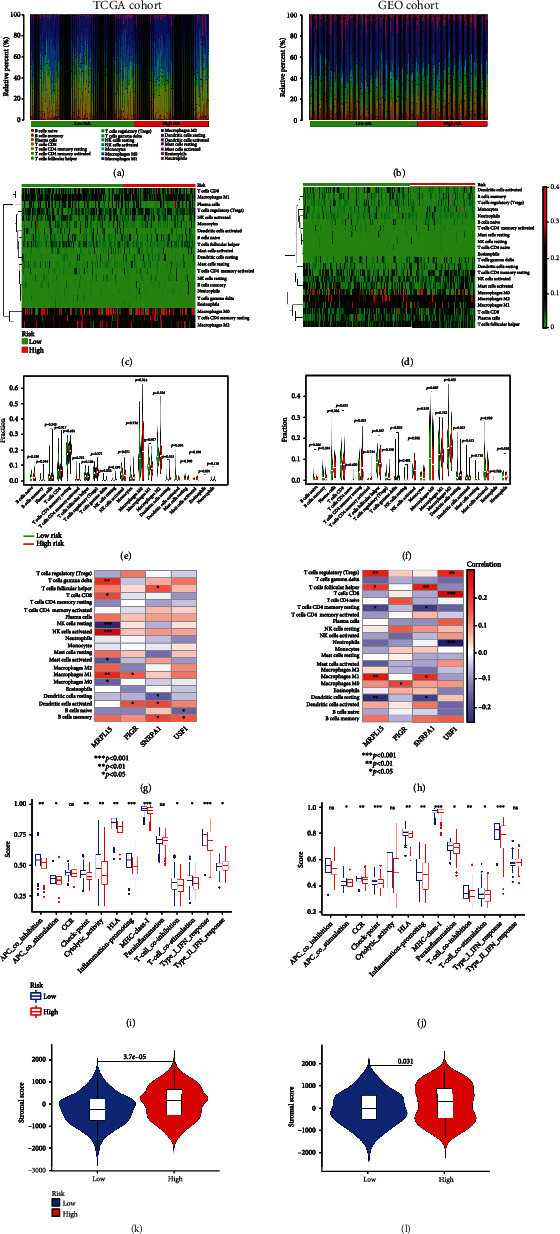
Analysis of the connection between tumor microenvironment and risk score in TCGA and GEO cohorts. (a, b) The relative proportion of immune-infiltrating cells in different risk groups. (c, d) Expression of each immune-infiltrating cell in different risk groups. (e, f) Comparison of immune-infiltrating cells in low-risk and high-risk groups. (g, h) Correlation between four important prognostic genes and immune-infiltrating cells. (i, j) Comparison of ssGSEA scores for 13 immune-related functions between high-risk and low-risk groups. Adjusted *p* values are shown as ns: not significant; ^∗^*p* < 0.05; ^∗∗^*p* < 0.01; ^∗∗∗^*p* < 0.001. (k, l) Comparison of stromal scores between different risk groups.

**Figure 6:**
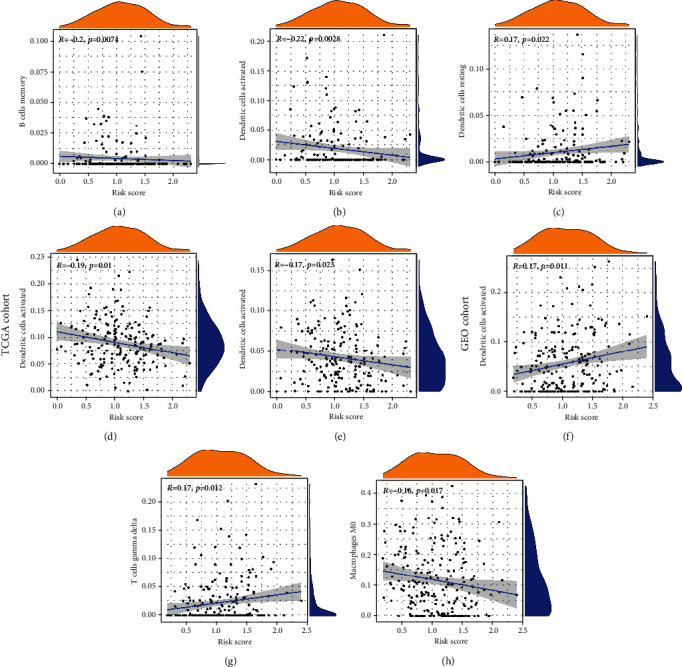
Correlation analysis of the risk score with infiltration abundances of several immune cells in TCGA cohorts (a–e) and GEO (f–h) cohorts.

The infiltration of immune cells was further assessed in each risk group. We obtained the proportions of 22 TIICs by the CIBERSORT algorithm (Figures 5(a) and 5(b)) and presented the expression of each immune-infiltrating cell across different groups (Figures 5(c) and 5(d)). As was displayed in Figures 5(e) and 5(f), memory B cells, M1 macrophages, resting dendritic cells, and activated dendritic cells differed significantly between the two risk groups in the TCGA cohort, while CD4^+^ resting memory T cells, gamma delta T cells, and resting dendritic cells differed significantly between the two risk groups in the GEO cohort. Furthermore, correlations between immune-infiltrating cells and four prognostic genes were analyzed in both cohorts (Figures 5(g) and 5(h)). We selected significant immune cells from the correlation results and explored the relationship between the ERGRS and infiltration levels of immune cells. For the TCGA cohort, the ERGRS had a positive association with infiltration levels of resting dendritic cells (*p* = 0.022) and was negatively correlated with memory B cells (*p* = 0.0074), M1 macrophage (*p* = 0.01), activated dendritic cells (*p* =0.0028), and activated NK cells (*p* = 0.023) (Figures 6(a)–6(e)). In the GEO cohort, however, risk scores were positively correlated with infiltration levels of CD4^+^ resting memory T cells (*p* = 0.011) and gamma delta T cells (*p* = 0.012) and had a negative association with infiltration levels of M0 macrophages (*p* = 0.017) (Figures 6(f)–6(h)).

Afterwards, to study the connections between the ERGRS and immune cell function, the activity of 13 immune-related pathways was assessed by ssGSEA analysis. In the TCGA cohort, immune function scores such as inflammation-promoting, MHC class I, and type I IFN response were significantly improved in patients with low ERGRSs. For the GEO cohort, the immune function scores such as check-point, MHC class I, and type I IFN response were observably increased in patients with low ERGRSs (Figures 5(i) and 5(j)). Finally, significant differences in stromal scores between different risk groups were found by the ESTIMATE algorithm, indicating that the proportion of stromal cells was related to the risk score (Figures 5(k) and 5(l)). Combined with the above results, we discovered that the ERGRS was connected with immune cell infiltration, suggesting that targeting ERGs had a regulatory effect on TIME in OV patients.

### 3.6. The Function of Risk Score in Forecasting the Response of Immunotherapy

**Figure 7:**
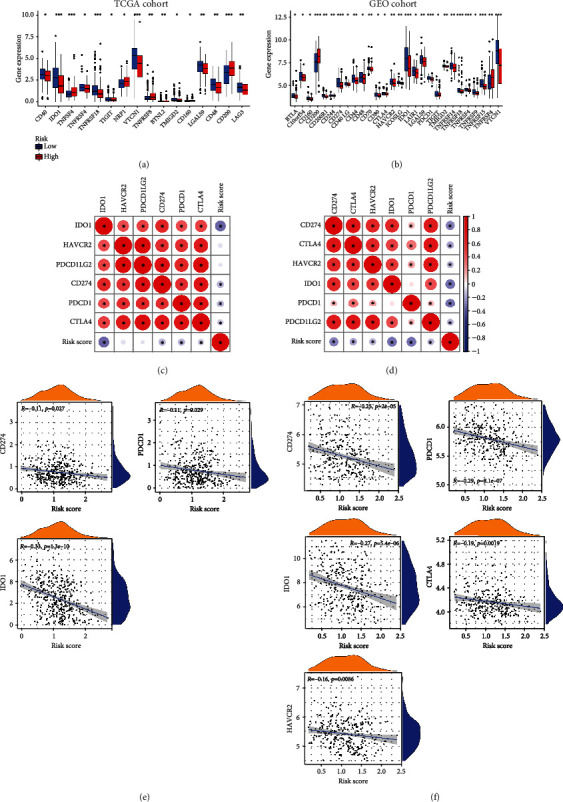
The connections between risk score and expression of immune checkpoint genes. (a, b) Expression levels of immune checkpoint genes in different risk groups in the TCGA and GEO cohorts. Adjusted *p* values are shown as ns: not significant; ^∗^*p* < 0.05; ^∗∗^*p* < 0.01; ^∗∗∗^*p* < 0.001. (c, d) Heat maps of the correlations between risk score and expression of six immune checkpoint inhibitor targets in the TCGA and GEO cohorts. The “^∗^” represents the statistically significant *p* value (*p* < 0.05). (e, f) Correlation analysis between risk score and expression of immune checkpoint inhibitor targets expression in the TCGA and GEO cohorts.

**Figure 8:**
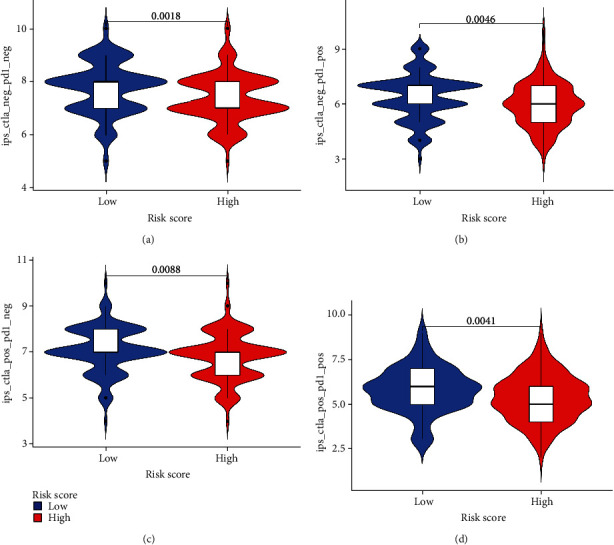
Predictive role of risk scores for different responses to immunotherapy strategies. (a) Without immunotherapy, high-risk scores resulted in poor prognosis compared with low-risk scores (*p* = 0.0018). (b) If only anti-PD1 immunotherapy was conducted, the group with higher risk scores had a poorer therapeutic effect than the group with lower risk scores (*p* = 0.0046). (c) If only anti-CTLA4 immunotherapy was used, the high-risk group had a worse prognosis compared with the low-risk group (*p* = 0.0088). (d) When anti-PD1 and anti-CTLA4 immunotherapies were used simultaneously, there was a significantly better prognosis in the low-risk group than in the high-risk group (*p* = 0.0041).

We studied the expression of genes for immune checkpoints in different risk patients. The results demonstrated that regardless of the TCGA cohort or the GEO cohort, the expression of genes for immune checkpoints differed between different groups (Figures 7(a) and 7(b)). Next, we further explored the connections between the ERGRS and the expression of six common targets of ICIs, including CD274 (PD-L1), PDCD1 (PD-1), PDCD1LG2, CTLA4, HAVCR2, and IDO1 (Figures 7(c) and 7(d)). The expression of CD274 (PD-L1), PDCD1 (PD-1), and IDO1 was negatively associated with risk scores in the TCGA dataset (*p* < 0.05, Figure 7(e)), while the expression of CD274 (PD-L1), PDCD1 (PD-1), CTLA4, HAVCR2, and IDO1 was negatively related to risk scores in the GEO dataset (*p* < 0.05, Figure 7(f)). Afterwards, two targets of ICIs (CTLA4 and PD-1) closely related to risk scores were chosen, and four immunotherapy strategies were evaluated in both risk groups by IPS. The IPSs of the four immunotherapies in patients with high risk were lower than in patients with low risk (Figures 8(a)–8(d)), which implied that patients with low-risk scores would benefit more from immunotherapy. The above outcomes suggested that the ERGRS could forecast the effect of immunotherapy to guide the selection of immunotherapy strategies.

### 3.7. Copy Number Variation Analysis

**Figure 9:**
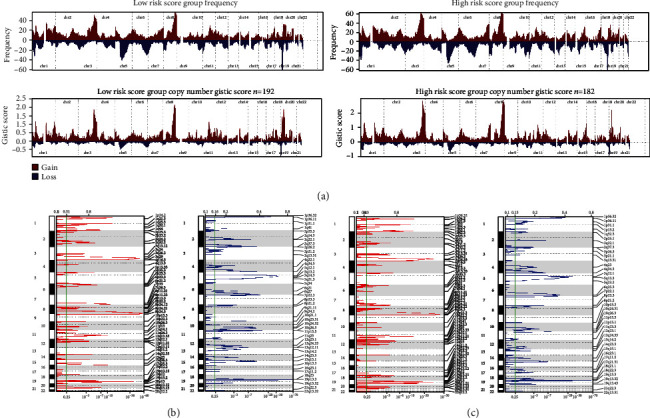
Copy number variation analysis. (a) Copy number profiles for low-risk and high-risk groups based on frequency and amplitude, with gains in red and losses in blue. Gene segments were placed according to their location on chromosomes, ranging from chromosome 1 to chromosome 22. (b) Regions of focal amplification (red) and focal deletion (blue) in the low-risk group were delineated by GISTIC2.0 software. (c) Regions of focal amplification (red) and focal deletion (blue) in the high-risk group were delineated by GISTIC2.0 software. *q* value (bottom) and *G*-score (Top) were listed as *x*-axis. The green line indicated the cutoff value of the *q* value (0.25). Chromosome numbers were labeled on the left and regions with recurrent copy number variation that was labeled on the right of each plot.

Because of the association between copy number variation and disease [32], we further explored copy number variations between different risk groups.  Figure 9(a) shows the distribution of the *G*-score for all chromosomes in both risk groups. Focal amplification and deletion of different chromosomal regions were found in both risk groups (Figures 9(b) and 9(c)). We discovered that the group with high-risk scores had more regions of amplification and deletion than the group with low-risk scores. These results suggested that patients with high risk had relatively lower immunogenicity than patients with low risk.

### 3.8. Drug Sensitivity Analysis of Independent Prognostic ERGs

**Figure 10:**
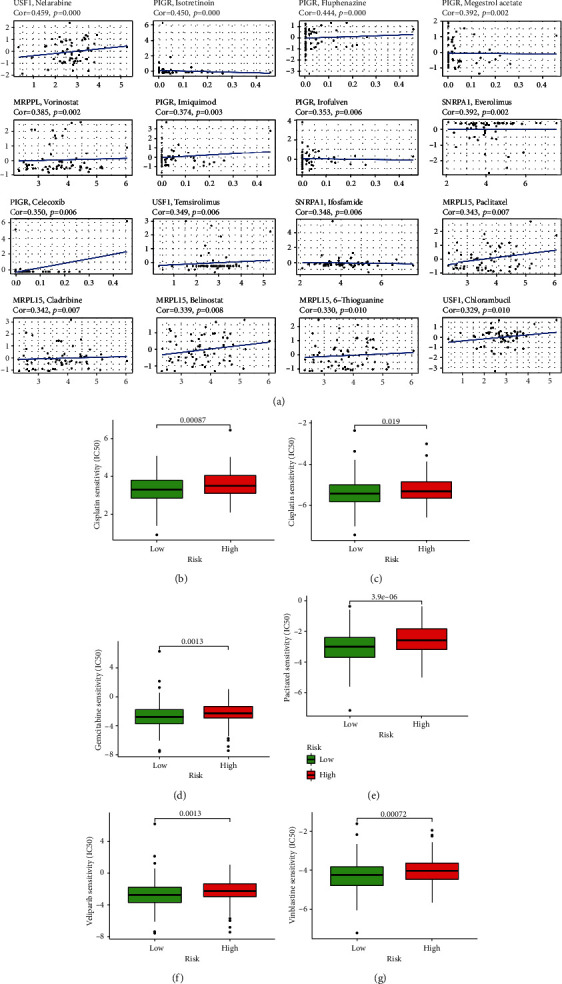
Drug sensitivity analysis of independent prognostic genes. (a) Four independent prognostic genes and drug sensitivity (measured by *Z*-score) were analyzed by Pearson's correlation analysis. The top 16 associations were shown, ordered by *p* value (∣Pearson′s correlation | >0.25 and *p* < 0.05). (b–g) The significance of the difference in IC50 scores between high-risk and low-risk groups for 6 drugs, including cisplatin, docetaxel, gemcitabine, paclitaxel, veliparib, and vinblastine, was analyzed by Wilcoxon's test.

The drug sensitivity was assessed by the *Z*-score, with higher scores indicating greater sensitivity to drug treatment. Pearson correlation analysis was performed on the expression of four exosome-associated prognostic genes and the sensitivity of 216 FDA-approved drugs using NCI-60 cell line data in the CellMiner database.  Figure 10(a) reveals the top 16 significant associations between the expression of four ERGs and drug sensitivity. In addition, the connections between different risk groups and IC50 values of six drugs were also analyzed through the GDSC database. The results displayed that IC50 values for all six drugs differed significantly between different subgroups (*p* < 0.05, Figures 10(b)–10(g)), and the subgroup with low risk was more sensitive to the six drugs. The above results suggested that the ERGRM might be useful in predicting chemical sensitivity.

## 4. Discussion

The exosome is an extracellular vesicle composed of specific proteins, lipids, RNA, and DNA that deliver a payload of proteins and nucleic acids to recipient cells, which mediates information exchange between cells [33]. With the further study of the role and function of exosomes, we found that exosomes are of great importance to tumor genesis, growth, apoptosis, immune response, and chemoresistance in cancer [10, 16, 34, 35]. Many studies also demonstrated that exosomes have great potential in diagnosing and treating early malignant tumors [36, 37]. In addition, since exosomes are vital to the entire progression of OV [38–40], we could consider exosomes as new biomarkers and targets for OV and use ERGs to establish a model to evaluate patients' prognosis. In our study, through a series of regression analyses, we included four ERGs with predictive values into the model and established the corresponding risk model. Afterwards, OV patients were split into low-risk and high-risk groups, and we discovered that low-risk patients had better prognosis and higher survival rates than high-risk patients. It was verified that the ERGRM had good predictive power by the ROC curve. Besides, a study demonstrated that BRCA1 was a tumor suppressor gene with the mutated phenotype predisposed to breast and ovarian cancer [41]. Hence, we regarded the BRCA1 type as a clinical feature and explored the connections between the ERGRS and various clinical features. Subsequently, the results of univariate, multivariate Cox regression and ROC curves illustrated that the ERGRS, age, and tumor stage were importantly independent predictors for OV patients. Moreover, we discovered that the genome-wide group with low risk was enriched in signaling pathways related to immune factors through GSEA.

Exosomes in tumors are closely related to antigenic immune responses, and exosomes can pass MHC-peptide complexes to specific T cells to initiate adaptive immune responses [13]. Besides, exosomes can also mediate immunosuppression of tumor host cells, including inhibiting the hyperplasia of CD4^+^ and CD8^+^ T lymphocytes, inhibiting the cytotoxicity of NK cells, making macrophages activated in tumor invasion and metastasis, and facilitating the differentiation of regulatory T lymphocytes [16]. The TIME and infiltration of immune cells are related to cancer development, prognosis, and response to therapy [42]. Studies reported that tumor-infiltrating lymphocytes (TILs) accumulated in OV predicted a higher survival rate, and that TILs had a good prognostic value [43–46]. On the contrary, the existence of immunosuppressive regulatory T cells was related to the reduced OV survival rate [47–49]. In our study, we obtained the ratios of 22 TIICs using the CIBERSORT algorithm and compared the differences in various TIICs in different groups. Afterwards, we assessed the connections between the ERGRS and immune cell function by ssGSEA analysis, which showed that immune function scores of type I IFN response and MHC class I increased significantly in the group with low risk in both cohorts. According to the report, miR-146a in exosomes inhibited type I interferon responses in target cells, thus promoting viral replication [50]. Through these methods, we found a certain relationship between TIME and ERGRS.

Redon et al. found 1447 copy number variation regions covering 12% of the human genome in 270 healthy individuals in the HapMap project [51]. This suggested that more of the human genome is affected by copy number variation. Significant variations in copy number are often detrimental, and some studies revealed that copy number variation is associated with cancer progression and contributes to cancer susceptibility [32, 52, 53]. Therefore, we performed an analysis of copy number variation between different groups. Results indicated that the group with higher risk scores had more genomic amplifications and deletions than the group with low risk. This manifested that the group with high risk had lower immunogenicity.

Various types of tumors lead to immune evasion by expressing immune checkpoints, so ICIs are crucial to immunotherapy. PD-1, PD-L1, and CTLA-4 inhibitors display encouraging therapeutic effects among common ICIs, some of which have been ratified to treat melanoma, non-small-cell lung cancer, kidney cancer, and bladder cancer [54–56]. According to the study, upregulation of HAVCR in renal cell carcinoma may stand for a new mechanism to stimulate tumor progression and angiogenesis, and HAVCR is linked to patients' prognosis [57]. However, the high expression of PD-L1 was also considered connected with a favorable prognosis in OV [58]. In addition, IDO1 is a significant immune-related gene in female cancers, and the high expression of IDO1 is connected with good prognosis in breast and ovarian cancer [59]. Similar to PD-L1, the study on the expression of IDO1 in OV came to different conclusions [60]. It may be that they are strongly correlated with immune cell populations, and activated T cells touch off negative feedback mechanisms in TIME, which leads to immune homeostasis [59]. Our study revealed that the expression of CD274 (PD-L1), PDCD1 (PD-1), and IDO1 was all negatively related to the ERGRS. This showed the good prognostic ability of exosomes. According to studies, although ICIs have successfully treated other malignant tumors, the clinical application of checkpoint inhibitors in OV has been almost unsuccessful so far [20, 61]. Therefore, the therapeutic effect of ICIs in OV needs to be further explored. In the study, we selected two targets of ICI (CTLA4 and PD-1) that were greatly related to risk scores and used IPS to evaluate the connections between the ERGRS and immunotherapy response. The results suggested that the ERGRS was a good predictor of immunotherapeutic efficacy.

Although ICIs are a promising approach for immunotherapy, clinical application of ICIs in OV has rarely achieved satisfactory results. Therefore, the combination of immunotherapy with other treatments is essential [20], and the current standard of treatment for OV is still surgery and platinum-based chemotherapy. Chemotherapy with platinum drugs and paclitaxel is considered the first-line therapy for OV. Still, there was also a clinical trial suggesting that docetaxel-carboplatin represented an alternative first-line chemotherapy for patients with newly diagnosed OV [62]. One of the reasons for the survival rate in 5 years under 50% is that ovarian tumors often develop resistance to platinum-based drugs. This issue can be addressed as gemcitabine is an effective and safe drug in platinum-sensitive and resistant recurrent OV [63]. Additionally, an article reported that pregnant patients with malignant ovarian tumors could be effectively treated with cisplatin, vinblastine, and bleomycin [64]. Patients with lower ERGRS were more sensitive to cisplatin, docetaxel, gemcitabine, paclitaxel, veliparib, and vinblastine in our study, which analyzed the predictive effect of the risk model for chemosensitivity.

This study also has some limitations. First of all, all analyzed samples are from public datasets, as are needed for further verification. Moreover, the risk model mainly considers only ERGs, and it is difficult to explain the specific association between exosomes and TIME. Therefore, extensive multicenter clinical trials are also needed to support our hypothesis and thus provide new insights into immunotherapy for OV patients.

## 5. Conclusions

We established and validated an ERGRM connected with immune infiltration to forecast the prognosis of OV patients, which can be used as an independent predictor and guide future immunotherapy strategies.

## Data Availability

The data used to support the findings of this study are available from the corresponding authors on reasonable request.

## Supplementary Materials

Supplementary Materials Supplementary Table 1: univariate and multivariate Cox regression analyses of the prognosis-related factors. Supplementary Figure 1: Venn diagram of genes in the TCGA cohort and ERGs. 117 related to ERGs in the TCGA cohort were ascertained. Supplementary Figure 2: selection of ERGs associated with patient prognosis by LASSO regression analysis. (A) The LASSO coefficient profiles of the five genes screened by univariate Cox regression analysis are given. (B) The optimal value of the penalty parameter *λ* is determined by tenfold crossvalidation results. Supplementary Figure 3: the prognostic risk model for evaluating patients was constructed by multivariate Cox proportional hazards regression analysis. Supplementary Figure 4: the connections between the risk score and various clinical characteristics. (A) Difference analysis of the risk score between different survival states. (B) Difference analysis of the risk score between different ages. (C) Difference analysis of the risk score between different tumor stages. (D) Difference analysis of the risk score between different tumor grades. (E) Difference analysis of the risk score between different therapy types. (F) Difference analysis of the risk score between different BRCA1 types. Supplementary Figure 5: assessment of the prognostic power of risk scores and other clinical characteristics. (A, B) Areas under the ROC curve for risk scores and other clinical characteristics in the TCGA and GEO cohorts. (C, D) In the TCGA and GEO cohorts, the reliability of the combination of risk scores and other clinical characteristics was assessed by comparing the AUC values of risk scores, clinical factors, and combinations of clinical factors and risk scores. Supplementary Figure 6: GESA of high- and low-risk groups. (A) The enriched pathways of the high-risk group. (B) The enriched pathways of the low-risk group.
