# Supplementary material for: Exosome-Associated Gene Signature for Predicting the Prognosis of Ovarian Cancer Patients
Source: J Immunol Res. 2023 Jan 23;2023:8727884. doi: 10.1155/2023/8727884 (PMC9886487; doi:10.1155/2023/8727884)
Supplement: Supplementary Materials — Supplementary Table 1: univariate and multivariate Cox regression analyses of the prognosis-related factors. Supplementary Figure 1: Venn diagram of genes in the TCGA cohort and ERGs. 117 related to ERGs in the TCGA cohort were ascertained. Supplementary Figure 2: selection of ERGs associated with patient prognosis by LASSO regression analysis. (A) The LASSO coefficient profiles of the five genes screened by univariate Cox regression analysis are given. (B) The optimal value of the penalty parameter λ is determined by tenfold crossvalidation results. Supplementary Figure 3: the prognostic risk model for evaluating patients was constructed by multivariate Cox proportional hazards regression analysis. Supplementary Figure 4: the connections between the risk score and various clinical characteristics. (A) Difference analysis of the risk score between different survival states. (B) Difference analysis of the risk score between different ages. (C) Difference analysis of the risk score between different tumor stages. (D) Difference analysis of the risk score between different tumor grades. (E) Difference analysis of the risk score between different therapy types. (F) Difference analysis of the risk score between different BRCA1 types. Supplementary Figure 5: assessment of the prognostic power of risk scores and other clinical characteristics. (A, B) Areas under the ROC curve for risk scores and other clinical characteristics in the TCGA and GEO cohorts. (C, D) In the TCGA and GEO cohorts, the reliability of the combination of risk scores and other clinical characteristics was assessed by comparing the AUC values of risk scores, clinical factors, and combinations of clinical factors and risk scores. Supplementary Figure 6: GESA of high- and low-risk groups. (A) The enriched pathways of the high-risk group. (B) The enriched pathways of the low-risk group. [file 8727884.f1.docx]

**Supplementary Materials**

**Supplementary Table 1** Univariate and multivariate Cox regression analyses of the prognosis-related factors.

| Variables | Univariate analysis | | | Multivariate analysis | | |
| --- | --- | --- | --- | --- | --- | --- |
|  | **HR** | **95%CI** | **P-value** | **HR** | **95%CI** | **P-value** |
| TCGA training set |  |  |  |  |  |  |
| age | 1.270 | 0.880-1.833 | 0.202 | 1.299 | 0.897-1.882 | 0.167 |
| grade | 1.855 | 0.967-3.557 | 0.063 | 2.007 | 1.015-3.969 | 0.045 |
| stage | 2.610 | 0.827-8.231 | 0.102 | 1.955 | 0.594-6.430 | 0.270 |
| risk score | 2.342 | 1.606-3.414 | <0.001 | 2.419 | 1.659-3.528 | <0.001 |
| TCGA testing set |  |  |  |  |  |  |
| age | 1.593 | 1.085-2.341 | 0.018 | 1.605 | 1.081-2.382 | 0.019 |
| grade | 0.815 | 0.483-1.374 | 0.442 | 0.679 | 0.396-1.162 | 0.158 |
| stage | 2.214 | 0.544-9.008 | 0.267 | 2.019 | 0.491-8.300 | 0.330 |
| risk score | 1.323 | 0.866-2.019 | 0.195 | 1.271 | 0.832-1.944 | 0.268 |
| Entire TCGA dataset |  |  |  |  |  |  |
| age | 1.405 | 1.078-1.832 | 0.012 | 1.342 | 1.028-1.752 | 0.030 |
| grade | 1.182 | 0.788-1.770 | 0.419 | 1.079 | 0.715-1.630 | 0.717 |
| stage | 2.418 | 0.995-5.877 | 0.051 | 2.278 | 0.922-5.626 | 0.074 |
| risk score | 1.816 | 1.369-2.410 | <0.001 | 1.750 | 1.319-2.323 | <0.001 |
| GEO dataset |  |  |  |  |  |  |
| age | 1.563 | 1.074-2.273 | 0.020 | 1.591 | 1.088-2.326 | 0.017 |
| grade | 1.302 | 0.882-1.924 | 0.184 | 1.073 | 0.719-1.603 | 0.730 |
| stage | 7.006 | 2.221-22.098 | <0.001 | 6.288 | 1.970-20.077 | 0.002 |
| risk score | 1.851 | 1.208-2.835 | 0.004 | 1.820 | 1.168-2.834 | 0.008 |


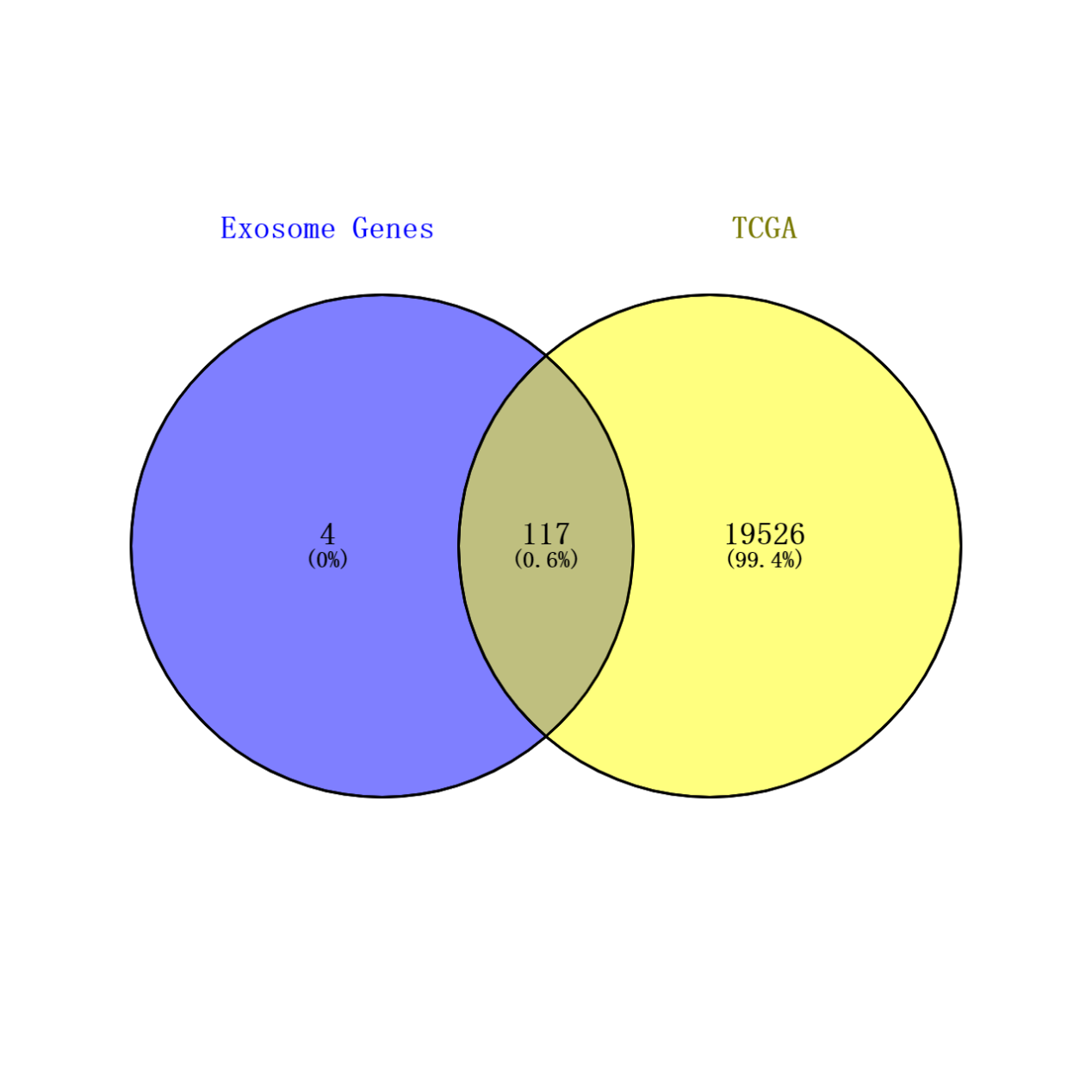


**Supplementary Figure 1** Venn diagram of genes in the TCGA cohort and ERGs. 117 related to ERGs in the TCGA cohort were ascertained.


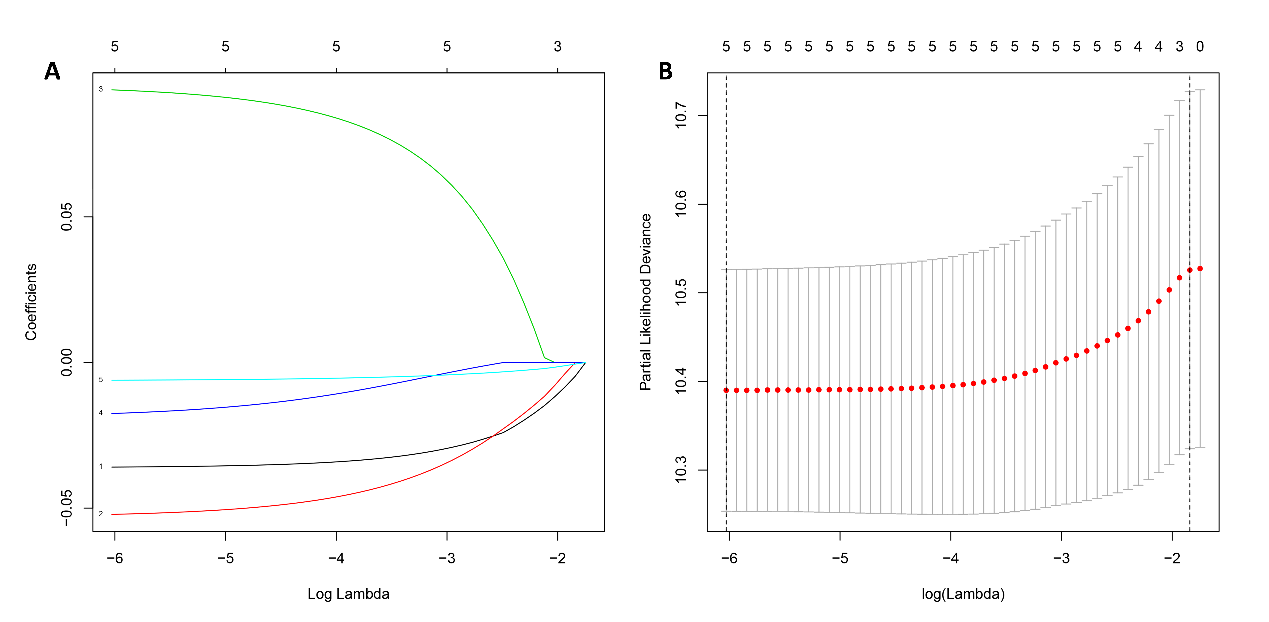


**Supplementary Figure 2** Selection of ERGs associated with patient prognosis by LASSO regression analysis. (A) The LASSO coefficient profiles of the five genes screened by univariate Cox regression analysis are given. (B) The optimal value of the penalty parameter λ is determined by tenfold cross-validations results.


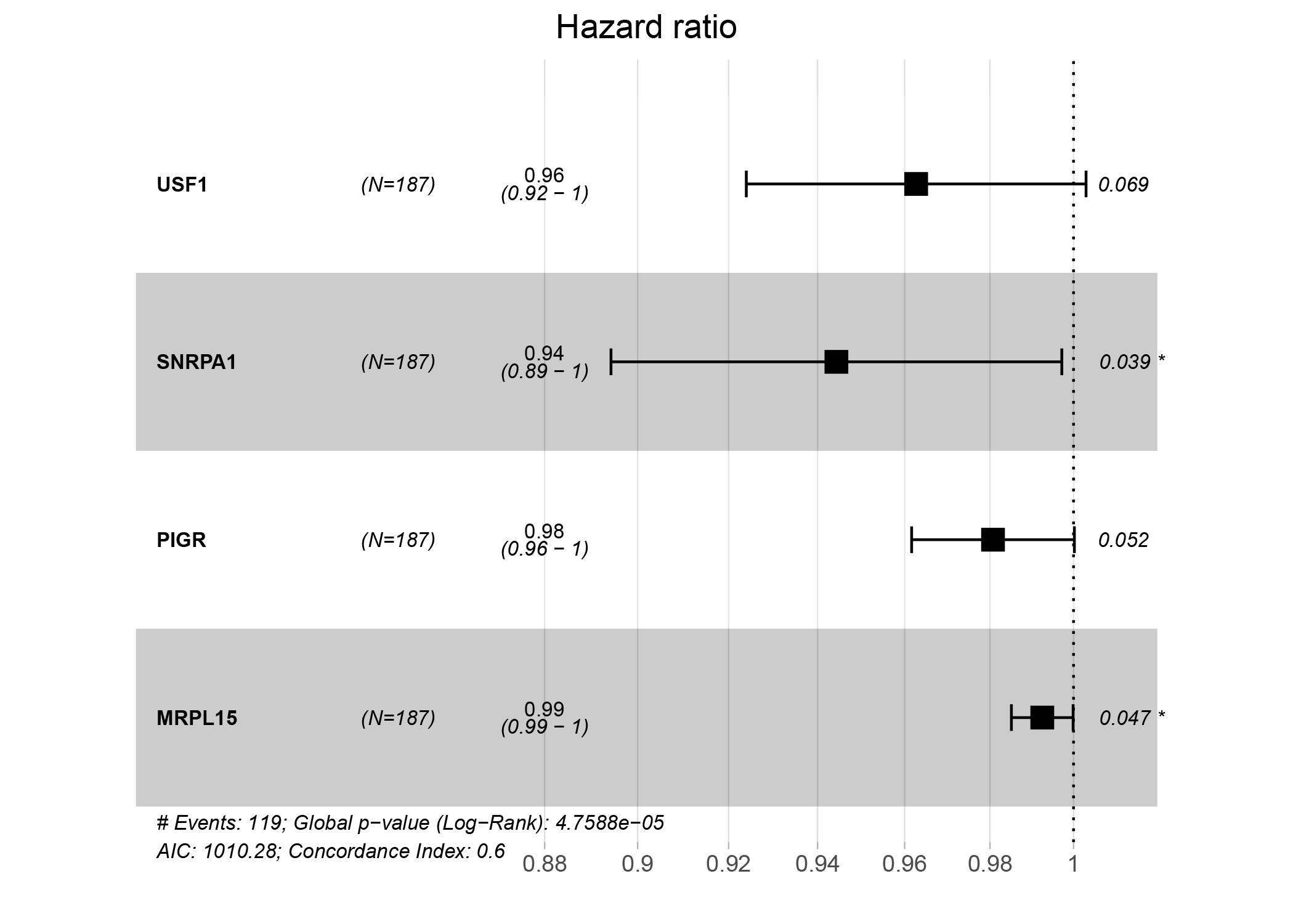


**Supplementary Figure 3** The prognostic risk model for evaluating patients was constructed by multivariate Cox proportional hazards regression analysis.


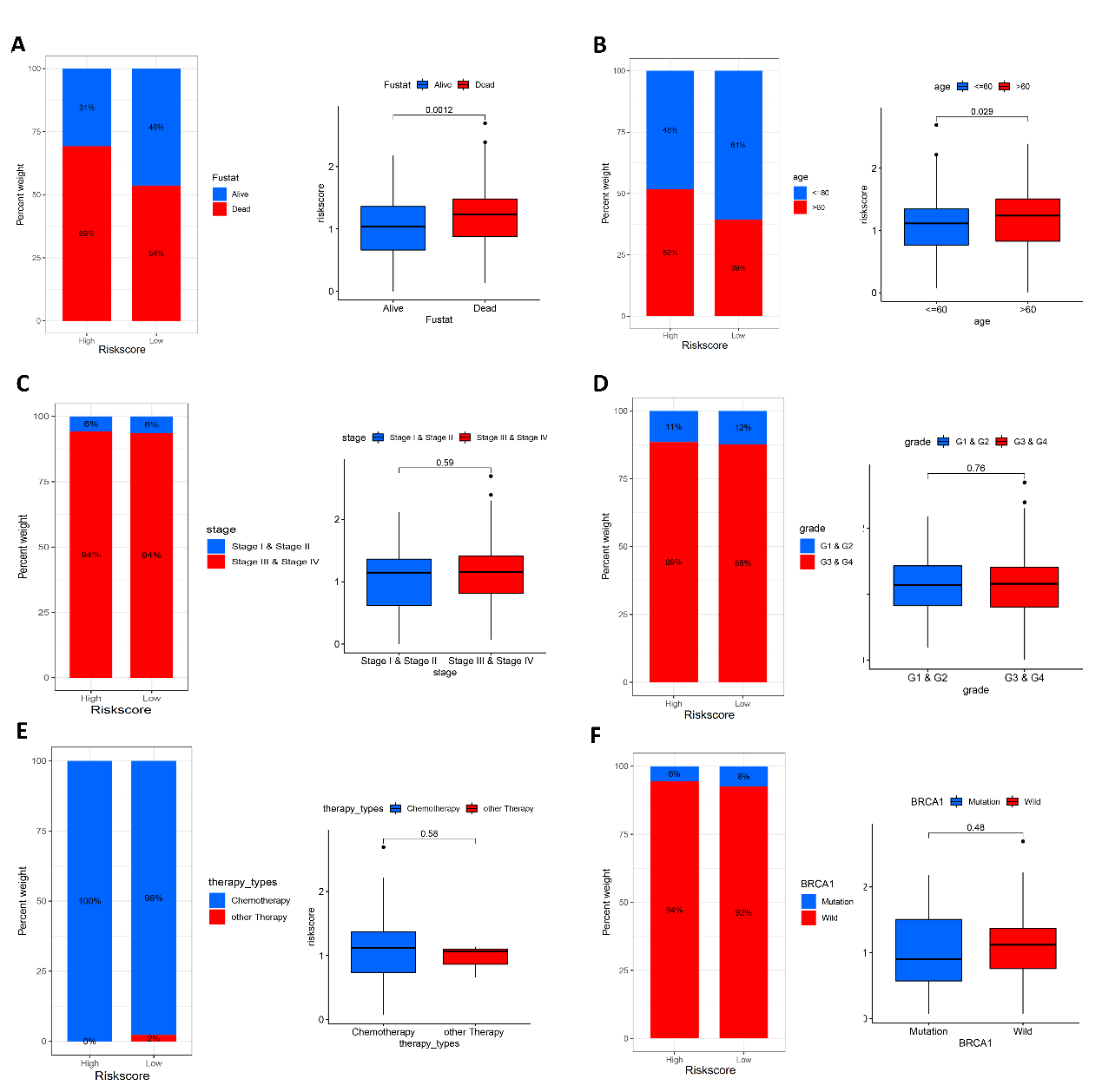


**Supplementary Figure 4** The connections between the risk score and various clinical characteristics. (A) Difference analysis of the risk score between different survival states. (B) Difference analysis of the risk score between different ages. (C) Difference analysis of the risk score between different tumor stages. (D) Difference analysis of the risk score between different tumor grades. (E) Difference analysis of the risk score between different therapy types. (F) Difference analysis of the risk score between different BRCA1 types.


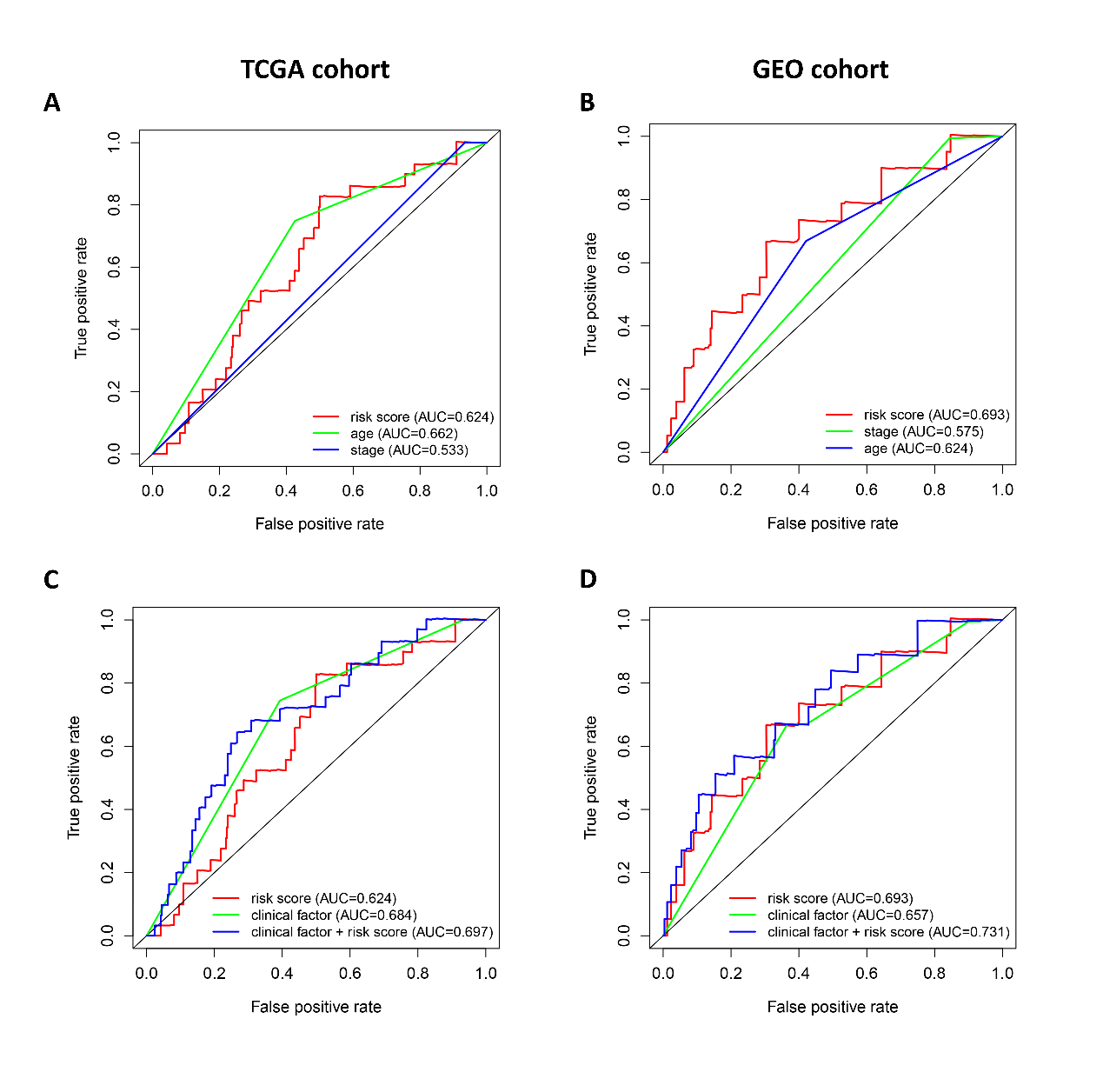


**Supplementary Figure 5** Assessment of the prognostic power of risk scores and other clinical characteristics. (A-B) Areas under the ROC curve for risk scores and other clinical characteristics in ​​the TCGA and GEO cohorts. (C-D) In ​​the TCGA and GEO cohorts, the reliability of the combination of risk scores and other clinical characteristics was assessed by comparing the AUC values ​​of risk scores, clinical factors, and combinations of clinical factors and risk scores.


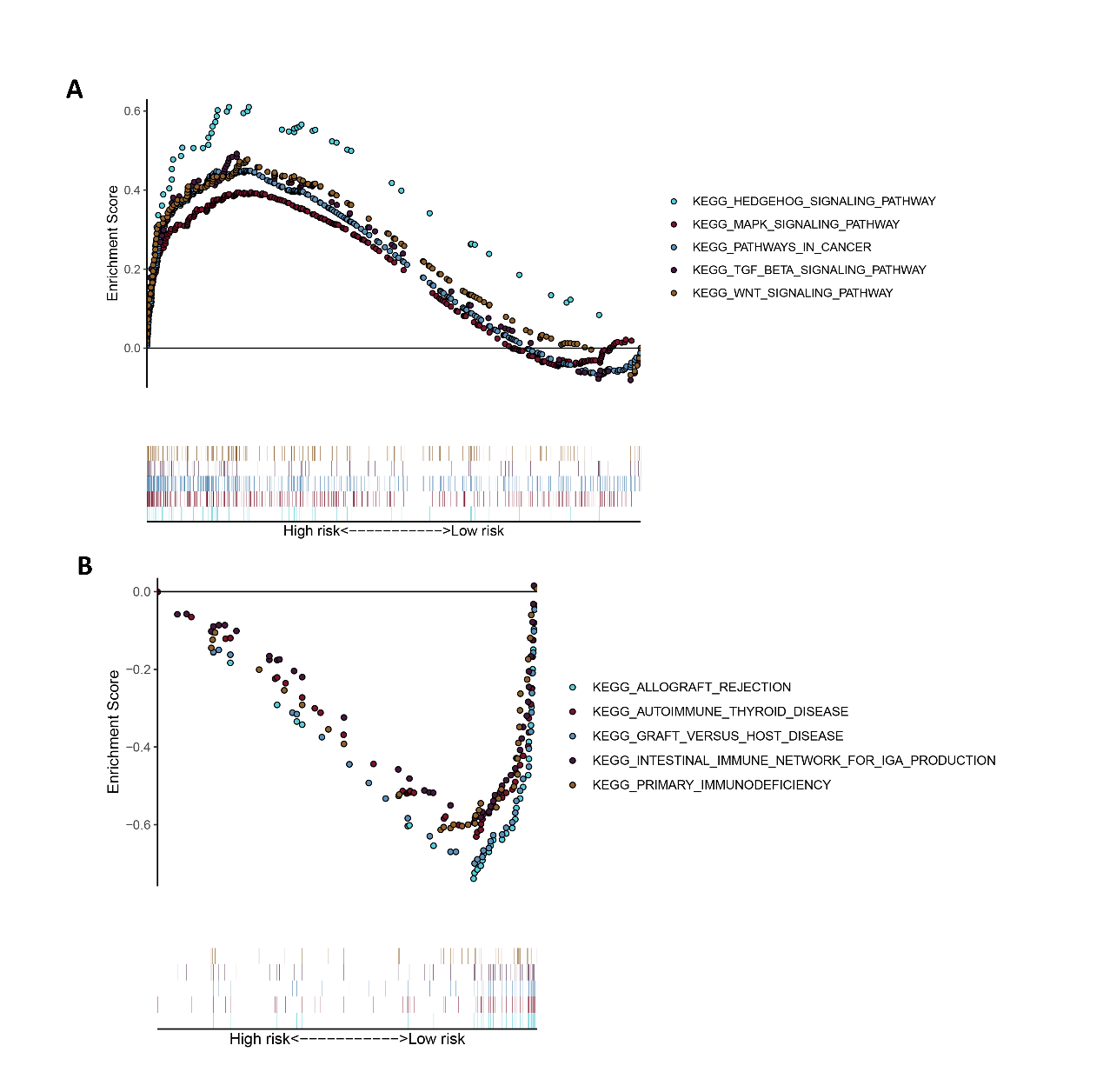


**Supplementary Figure 6** GESA of high-and low-risk groups. (A) The enriched pathways of the high-risk group. (B) The enriched pathways of the low-risk group.
